# A case of thyroid hormone resistance with coincidental pituitary macroadenoma

**DOI:** 10.1210/jcemcr/luag051

**Published:** 2026-04-08

**Authors:** Yi-An Pan, Liyan Wang, Usman Hammawa Malabu, Swetha Rangaswamaiah

**Affiliations:** Department of Endocrinology and Diabetes, Townsville University Hospital, Townsville, Queensland 4814, Australia; Department of Endocrinology and Diabetes, Townsville University Hospital, Townsville, Queensland 4814, Australia; Department of Endocrinology and Diabetes, Townsville University Hospital, Townsville, Queensland 4814, Australia; Head of Translational Research in Endocrinology and Diabetes at the College of Medicine and Dentistry, James Cook University, Townsville, Queensland 4814, Australia; Department of Endocrinology and Diabetes, Townsville University Hospital, Townsville, Queensland 4814, Australia

**Keywords:** hypothalamic-pituitary, pituitary tumors/mass, thyroid, hyperthyroidism, thyroid laboratory testing, thyroid tumor/goiter/mass

## Abstract

Central hyperthyroidism can be a diagnostic challenge, and most commonly arises from a thyrotropin-secreting pituitary adenoma (TSHoma) or thyroid hormone resistance (RTH). Assay interference from heterophile antibodies can also produce spurious biochemical results that mimic these conditions. Accurate distinction between these entities is essential as management strategies differ substantially. We present the case of a 35-year-old man with central hyperthyroidism in the context of a large pituitary macroadenoma, initially suggestive of a TSHoma. Comprehensive biochemical evaluation, dynamic testing and subsequent genetic testing, identified a heterozygous autosomal dominant *THRB* pathogenic variant (Val458Ala), confirming the diagnosis of RTH and thereby avoiding unnecessary pituitary surgery.

## Introduction

The coexistence of thyroid hormone resistance (RTH) and pituitary adenoma is rare, and most reported cases involve microadenomas. We report the case of a 35-year-old man who presented with thyrotoxic symptoms, biochemical evidence of central hyperthyroidism, and a large 26-mm pituitary macroadenoma, findings strongly suggestive of a thyrotropin (TSH)-secreting pituitary adenoma (TSHoma). Interestingly, his childhood history of hearing impairment and mild speech impediment provided a subtle clue to an underlying genetic etiology. A systematic evaluation, including dynamic testing, ultimately established the diagnosis of RTH and thereby avoiding unnecessary pituitary surgery.

## Case presentation

A 35-year-old man presented with new-onset paranoid delusions following several months of retro-orbital headache, palpitations, tremors, and anxiety. He had no weight loss, gastrointestinal symptoms, or family history of endocrinopathies. Examination revealed sinus tachycardia at 130 beats per minute and a blood pressure of 124/82 mm Hg, with normal respiratory rate and oxygen saturation. He had mild bilateral hand tremors, but no goiter and no clinical features of acromegaly. Cardiorespiratory and abdominal examinations were unremarkable. He was evaluated by a psychiatrist, who noted a longstanding history of low mood and a history of childhood bullying related to his speech stutter that was attributed to congenital hearing impairment. He was started on olanzapine 10 mg thrice daily for acute psychosis and was admitted for further evaluation.

## Diagnostic assessment

**Figure 1:**
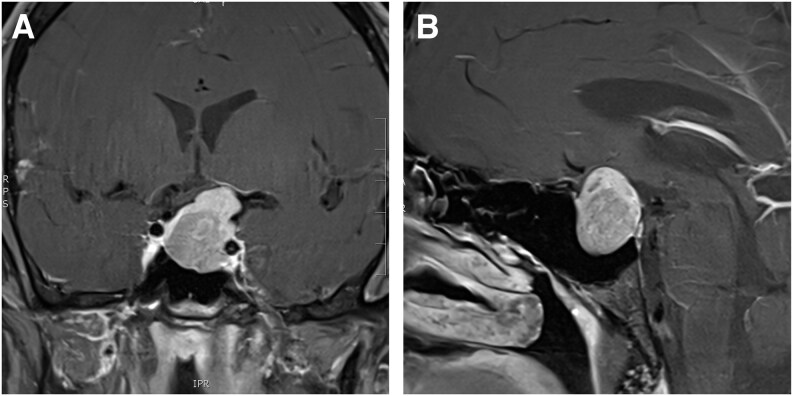
Magnetic resonance imaging (A) coronal (B) sagittal of the brain showing a 24.7 × 18.8 × 26.7-mm macroadenoma deviating the left optic nerve and chiasm.

During the patient’s organic psychiatric work-up, thyroid function test revealed central hyperthyroidism with a free thyroxine (T4) of 51 pmol/L (3.96 ng/dL; reference interval [RI], 11.5-22.7 pmol/L; 0.89-1.76 ng/dL), free triiodothyronine (T3) of 18.7 pmol/L (12.2 pg/mL; RI, 3.5-6.5 pmol/L; 2.3-4.2 pg/mL) with an unsuppressed TSH of 3.14 mU/L (RI, 0.55-4.78 mU/L), with negative TSH receptor antibodies. Following endocrinology consultation, further anterior pituitary work-up revealed an elevated insulin-like growth factor 1 (IGF-1) of 49 nmol/L (374 ng/mL; RI, 16-39 nmol/L; 113-297 ng/mL), and mildly elevated prolactin of 341 mIU/L (16.1 ng/mL; RI, 55-300 mIU/L; 2.6-14.2 ng/mL) secondary to stalk effect. The hypothalamic-pituitary-adrenal axis and hypothalamic-pituitary-gonadal axis were preserved (Table 1). Magnetic resonance imaging (MRI) of the brain revealed a pituitary macroadenoma measuring 24.7 × 18.8 × 26.7 mm (Fig. 1). The lesion caused mass effect with deviation of the left optic tract and chiasm as well as abutting the left cavernous internal carotid artery. Visual perimetry performed by an optometrist confirmed normal visual fields and acuity.

**Table 1:** Summary of the patient's anterior pituitary panel during the initial work of the pituitary macroadenoma

Hormone test	Values	Reference ranges
TSH	**3.14** **mU/L**	0.55-4.78 mU/L
Free T4	**51 pmol/L** (3.96 ng/dL)	11.5-22.7 pmol/L (0.89-1.76 ng/dL)
Free T3	**18.7 pmol/L** (12.2 pg/mL)	3.5-6.5 pmol/L (2.3-4.2 pg/mL)
IGF-1	**49 nmol/L** (374 ng/mL)	16-39 nmol/L (113-297 ng/mL)
Prolactin	**341 mIU/L** (16.1 ng/mL)	55-300 mIU/L (2.6-14.2 ng/mL)
ACTH	9.3 pmol/L (42 pg/mL)	2.2-13.2 pmol/L (10-60 pg/mL)
Cortisol	597 nmol/L (21.6 µg/dL)	137-689 nmol/L (5-25 µg/dL)
LH	4.2 IU/L	1.5-9.3 IU/L
FSH	5.5 IU/L	1.4-18 IU/L
Testosterone	21 nmol/L (605 ng/dL)	10.4-31.2 nmol/L (300-900 ng/dL)

Abnormal values are shown in bold font. Values in parenthesis are conventional units.

Abbreviations: ACTH, adrenocorticotropin; FSH, follicle-stimulating hormone; IGF-1, insulin-like growth factor 1; LH, luteinizing hormone; T3, triiodothyronine; T4, thyroxine; TSH, thyrotropin.

The differential diagnoses included TSHoma with growth hormone (GH) cosecretion, RTH, and heterophile antibody assay–interference. The patient was initiated on carbimazole 10 mg twice daily and propranolol 10 mg thrice daily for symptom control. Despite 2 months of carbimazole therapy, there was minimal change in thyroid function: free T4 of 46 pmol/L (3.57 ng/dL), free T3 of 16 pmol/L (10.41 pg/mL), and TSH of 10.43 mU/L. An oral glucose tolerance test (OGTT) showed normal GH suppression (GH level <1.0 µg/L post OGTT), suggesting an absence of autonomous GH secretion. Additional biochemical investigations revealed a borderline α-subunit level of 0.7 U/L (<0.7 U/L) and sex hormone–binding globulin (SHBG) of 19 nmol/L (1.8 μg/mL; RI, 10-60 nmol/L; 1.1-6.7 μg/mL) and a thyroid uptake scan showing diffuse uptake, making it challenging to distinguish between TSHoma and RTH. Heterophile antibody interference was ruled out by our chemical pathology laboratory. A thyrotropin-releasing hormone (TRH) stimulation test was performed that showed a 6-fold increase in TSH (TSH 10.43 mU/L pre test and TSH 64.07 mU/L post test), consistent with RTH (Table 2). Genetic testing revealed an autosomal dominant, heterozygous pathogenic variant of the *THRB* gene on chromosome 3 (c. 1373T>C; p. [Val458Ala]), confirming the diagnosis of RTH.

**Table 2:** Thyrotropin-releasing hormone stimulation test showing a 6-fold increase in thyrotropin in response to TRH stimulation

TRH stimulation		
Pretest	20 min	60 min
TSH: 10.43 mU/L	TSH: 64.07 mU/L	TSH: 39.94 mU/L

A greater than 4-fold increase is expected for thyroid hormone resistance, while a blunted response less than 2-fold increase is expected for TSHoma.

Abbreviations: TRH, thyrotropin-releasing hormone; TSH, thyrotropin.

## Treatment

After confirming RTH, carbimazole was discontinued and the patient remained on propranolol 10 mg thrice daily and olanzapine 20 mg daily for symptom control.

## Outcome and follow-up

Currently, the patient is under joint endocrinology and neurosurgery follow-up with pituitary MRI and visual perimetry visits every 6 months, with consideration of surgical debulking if his vision deteriorates. From a metabolic perspective, he is undergoing cardiovascular risk assessment and risk factor modification. He has also been referred to an endocrine geneticist for genetic counseling and family screening.

## Discussion

**Figure 2:**
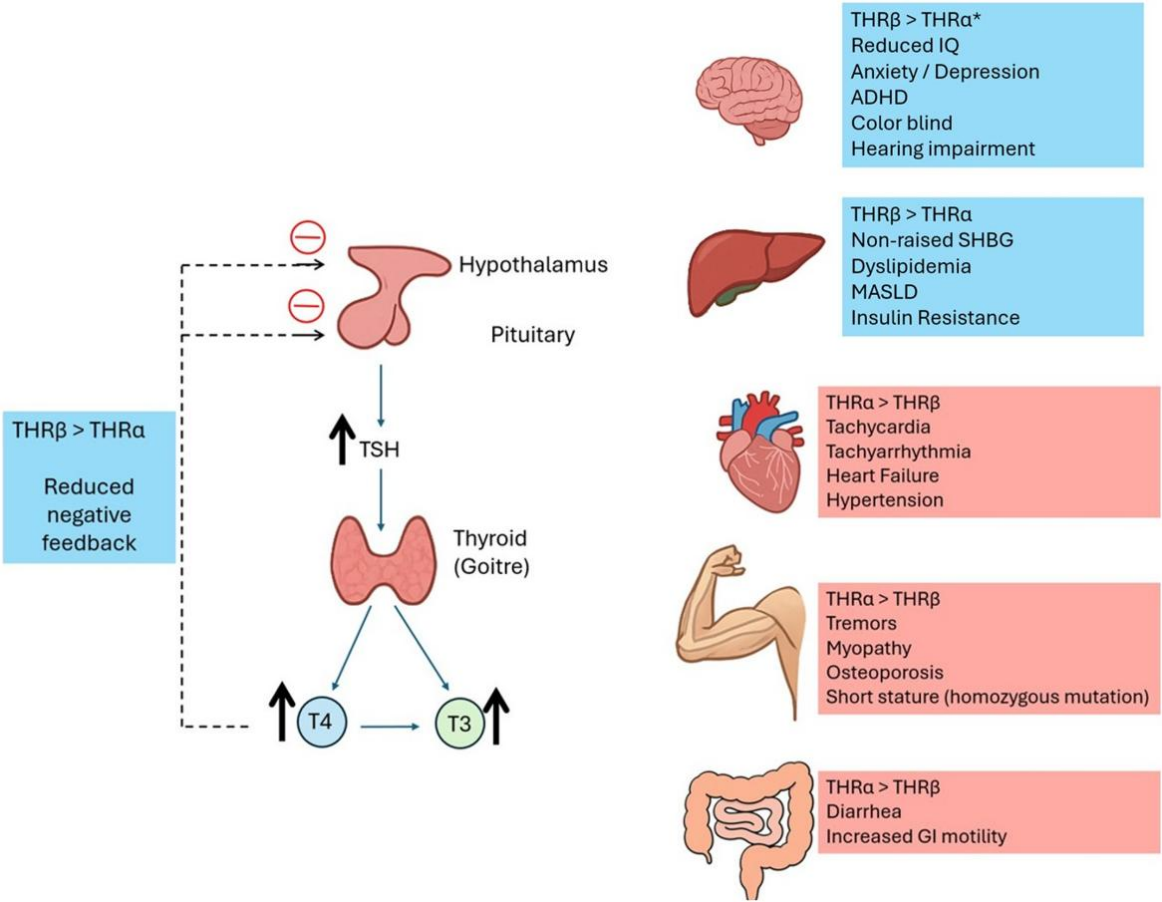
This diagram summaries the variable tissue distribution of thyroid hormone receptor (THR) β and THRα, which results in coexisting features of hyperthyroidism and hypothyroidism in carriers of THRβ mutation. Tissues abundant in mutated THRβ are resistant to thyroid hormone, leading to a hypothyroid state, while tissues abundant in wild-type THRα have normal response and are in a hyperthyroid state due to high circulating thyroxine (T4) and triiodothyronine (T3). The hypothalamic-pituitary system consists predominantly of THRβ resulting in impaired negative feedback signaling from thyroid hormone which leads to increased endogenous thyrotropin (TSH) production, goiter formation and hyperthyroid state. *While the brain predominantly expresses THRα, there are certain regions with higher THRβ expression that likely contribute to neurocognitive developmental delays and impaired hearing and visual development. Schematic has been developed with reference to *Moran & Chatterjee (2015) Resistance to thyroid hormone due to defective thyroid receptor alpha’, Best Practice & Research Clinical Endocrinology & Metabolism*.

RTH encompasses a group of rare inheritable disorders that affect peripheral tissue responsiveness to thyroid hormone. The most common cause of RTH is inheritable mutation of thyroid hormone receptor β (THRβ), which affects approximately 1 in every 40 000 live births [1]. THRβ mutation is characterized by elevated T4 and T3 with unsuppressed TSH, and variable clinical features ranging from minimal symptoms to clinical coexistence of hypothyroid and hyperthyroid states and, in severe cases, childhood cognitive impairment. Other rare causes of RTH include *THRα* mutation, MCT8 transporter mutation and *SBP2* or TRU-TCA1-1 mutation. THRs have 2 subtypes, THRα and THRβ, which are encoded by chromosome 17 and 3, respectively. Each receptor is further subdivided into 2 isoforms (THRα1/THRα2 and THRβ1/THRβ2), which have different tissue distribution and roles in the body. THRα is found mainly in the brain, bone, skeletal muscles, and cardiac myocytes, while THRβ is predominantly located in the hypothalamus, pituitary, liver, and kidney [2] (Fig. 2).

The majority of THRβ mutations are heterozygous (85%) and usually caused by single-nucleotide substitutions, resulting in an amino acid change that affects the T3 ligand–binding domain, reducing its affinity for T3 [3]. Approximately 85% of THRβ mutations originate from germline mutations and follow an autosomal dominant inheritance pattern. Rare frameshift mutations due to single-nucleotide deletions or insertions can lead to more severe receptor dysfunction that not only impairs T3 binding but also disrupts cofactor interactions, hence affecting downstream signaling and DNA binding [3]. The most severe clinical phenotypes of THRβ resistance are usually seen in patients with homozygous mutations or in extremely rare cases of THRβ gene deletion. The absence of functional THRβ results in dysmorphic features, short stature, mild to severe cognitive impairment, developmental delay, as well as deafness and color blindness due to the essential role of THRβ in cochlear and photoreceptor development in utero [4, 5]. All cases of THRβ resistance exhibit elevated T4 with unsuppressed TSH, reflecting resistant feedback on the hypothalamic-pituitary-thyroid axis due to mutated THRβ. Most patients have mild disease, often asymptomatic or presenting with a goiter and palpitations. Goiter is a manifestation of high circulating plasma TSH causing follicular cell hyperplasia. TSH also upregulates Na+/I– symporter and thyroid peroxidase activity, which increases iodine uptake, organification, and thus elevated T4 and T3 synthesis. Palpitations, tachyarrhythmia, and tremors, classic features of hyperthyroidism, are driven by the effects of elevated thyroid hormone on wild-type (WT) THRα on cardiac myocytes and skeletal muscles. Most individuals with heterozygous mutation have normal growth and neurocognitive development. However, mild learning disability, particularly affecting verbal performance with attention deficit hyperactivity disorder, may be present in some cases [6]. Children with more severe THRβ mutations (homozygous or gene deletion) are likely to develop more substantial cognitive impairment, which can be accompanied by color blindness, hearing loss, and delayed speech skills. They typically have dysmorphic features such as bird-like facies, goiter, pectus carinatum, stippled epiphyses, and developmental growth delay due to delayed bone maturation [4, 6, 7]. Our patient is an example of the mild, heterozygous phenotype. He is of normal stature and lacks dysmorphic features, but he does have a childhood hearing impairment that likely contributed to his paucity of speech into adulthood. The association between altered thyroid hormone levels and conditions such as depression, schizophrenia, and bipolar disorder is well recognized in patients with thyroid disease [8, 9]. Our patient's acute presentation with psychosis may reflect a neuropsychiatric vulnerability in carriers of pathogenic *THRβ* variants in the setting of chronically elevated circulating thyroid hormone levels.

Adults with THRβ resistance develop a unique metabolic profile due to heterogeneous tissue sensitivity to thyroid hormone. Tissues expressing predominantly WT THRα, such as bone, vascular smooth muscle, and skeletal muscle, are exposed to a relative state of thyrotoxicosis. Over time, this increases the risk of developing osteoporosis, systolic hypertension, and sarcopenia. In contrast, tissues predominantly expressing mutant THRβ, particularly the liver, exhibit a hypothyroid state despite elevated circulating thyroid hormone. This leads to dyslipidemia, particularly elevated low-density lipoprotein cholesterol. THRβ dysfunction has been implicated in the pathogenesis of metabolic dysfunction–associated steatotic liver disease (MASLD) [10]. THRβ resistance likely predisposes to hepatic steatosis, with a risk of progression to cirrhosis. These potential long-term metabolic abnormalities may contribute to an elevated cardiovascular risk profile, increased risk of insulin resistance and diabetes, and susceptibility to fragility fractures. Retrospective cohort studies of individuals with heterozygous THRβ mutations reported an increased incidence of earlier-onset major adverse cardiovascular events, occurring on average 11 years earlier compared to normal matched controls. This was associated with an estimated mean loss of 11 years of life expectancy in affected individuals, further supporting the long-term metabolic consequences of this disorder [11, 12].

The thyroid function pattern in THRβ resistance closely resembles that of a TSHoma. While MRI of the pituitary can aid in differentiation, the high prevalence of nonfunctional pituitary adenomas complicates clinical assessment. Plasma biomarkers can assist in distinguishing TSHoma from THRβ resistance. Notably, elevated SHBG and α-subunit levels are characteristic of TSHomas; however, they are observed in only 70% and 90% of cases, respectively [13]. The TRH stimulation test serves as a useful assessment of pituitary responsiveness to exogenous TRH (200 mcg). A blunted response, defined as a less than 4-fold increase, is seen in 90% of TSHomas [13]. A T3 suppression test can also aid in differentiating RTH from TSHoma, but its use is contraindicated in patients with known ischemic heart disease. Genetic testing is the best way to confirm the diagnosis of THRβ resistance, with 84% cases harboring an identifiable pathogenic variant [13]. Given its autosomal dominant inheritance pattern, family screening and genetic counseling for first-degree relatives should be considered based on current guidelines [14]. In patients with minimal symptoms, immunoassay interference by heterophile antibodies should be considered as a potential differential diagnosis. Rare cases of micro TSHomas that are difficult to detect on MRI have been reported. In these instances, ^11^C-methionine positron emission tomography–computed tomography image subtraction, in combination with somatostatin analogue therapy, has been used to unmask these tumors and facilitate surgical resection [15]. This case exemplifies the importance of plasma biomarker testing and dynamic endocrine testing to aid in establishing the correct diagnosis. Although his initial MRI and anterior pituitary profile were highly suggestive of a TSHoma, further evaluation led to the diagnosis of RTH instead. Limited availability and access to TRH and genetic testing in other centers may increase the likelihood of misdiagnosis and treatment.

The primary treatment of THRβ resistance focuses on managing symptoms of thyrotoxicosis, with β-blockers serving as first-line therapy for tachycardia and tremors. The medical management of symptomatic goiters in patients with RTH aims to suppress endogenous TSH secretion. This can be achieved with supraphysiologic doses of T3 (250 mcg) on alternate days, as reported in a single successful case report involving a young adult [16]. Alternatively, the thyromimetic agent 3,3,5-triiodothyroacetic acid (TRIAC) may be considered for TSH suppression but its use is limited by restricted availability in many countries [17]. Surgery remains a therapeutic option for patients with compressive goiters. However, it must be carefully considered given the operative risks and challenges of postthyroidectomy hormone replacement, since both TSH and free T4 are unreliable markers of treatment adequacy. Furthermore, case reports have described thyrotroph hyperplasia and pituitary enlargement in hypothyroid patients with RTH who are chronically underreplaced with levothyroxine [18, 19]. Beyond symptomatic management, clinicians should consider the potential long-term metabolic consequences of THRβ resistance. A proactive approach focused on risk factor surveillance may be beneficial in avoiding complications. Attention to nutritional optimization including adequate protein, calcium, and vitamin D intake may reduce the risk of sarcopenia and osteoporosis. Regular monitoring of blood pressure, lipid profile, and resting heart rate should be undertaken to assess the patient's cardiovascular risk profile. Screening for insulin resistance and monitoring for MASLD may aid in the prevention of or early detection of diabetes and cirrhosis.

Our case describes a patient with a large pituitary macroadenoma and a clinical presentation suggestive of TSHoma. Systematic evaluation and genetic testing instead led to the diagnosis of RTH, with his childhood hearing impairment serving as a clue to an underlying genetic syndrome. This case emphasizes the importance of a comprehensive work-up of patients with central hyperthyroidism, even those with pituitary macroadenomas, as misdiagnosis may result in unnecessary surgery.

## Learning points

- Patients presenting with central hyperthyroidism and a pituitary macroadenoma require systematic evaluation to exclude RTH or assay interference from heterophile antibodies, as misdiagnosis may lead to unnecessary surgery.
- The differentiation between RTH and TSHoma can be challenging. Normal SHBG and α-subunit levels support a diagnosis of RTH. Dynamic testing can provide further diagnostic precision. A normal TSH response to TRH stimulation (defined as a > 4-fold increase) or a complete to partial TSH suppression on the T3 suppression test both favor the diagnosis of RTH.
- Individuals with THRβ mutations carry an increased long-term risk of cardiovascular disease; ongoing monitoring and management of cardiovascular risk factors may improve long-term outcome.

## Contributors

All authors made individual contributions to authorship. Y.P., L.W., U.H.M., and S.R. were involved in the diagnosis and management of the patient as well as manuscript preparation and submission.

## Data Availability

Original data generated and analyzed for this case report are included in this published article.

## Abbreviations

GH: growth hormone
IGF-1: insulin-like growth factor 1
MASLD: metabolic dysfunction–associated steatotic liver disease
MRI: magnetic resonance imaging
OGTT: oral glucose tolerance test
RI: reference interval
RTH: thyroid hormone resistance
SHBG: sex hormone–binding globulin
T3: triiodothyronine
T4: thyroxine
TRH: thyrotropin-releasing hormone
TSH: thyrotropin
TSHoma: thyrotropin-secreting pituitary adenoma
WT: wild-type
